# Plethysmographic variability index and perfusion index in patients with axillary brachial plexus nerve catheters: An observational study

**DOI:** 10.1097/MD.0000000000035653

**Published:** 2023-10-20

**Authors:** Hale Aksu, Elvan Ocmen, Dilek Omur, Sezin Kizil, Ayse Karci

**Affiliations:** a Dept of Anesthesiology and Reanimation, School of Medicine, Dokuz Eylul University, Izmir, Turkey.

**Keywords:** axillary brachial plexus nerve, perfusion index, plethysmographic variability index

## Abstract

Axillary nerve blocks are commonly using for forearm and hand surgery. Especially for finger replacement it has been shown continuous plexus blockade improves microcirculation. Addition to that benefit continuous blockade provides adequate analgesia. In this study perfusion index (PI) and plethysmographic variability index (PVI) changes were used to evaluate in blocks success. The PVI and PI values were detected by a Radical-7TM finger pulse oximetry device (Massimo Corp, USA) in both fingers of 50 plastic surgery patients, who received an axillary brachial plexus catheter before surgery. Data recorded at baseline, during catheter replacement, after catheter replacement, and before surgery. All periods hemodynamic data, visual analog scala, Ramsey sedation score and patient satisfaction score were collected. In all 110 patients blocks were successfully applied, PI values in blocked arm increased after local analgesic application (during catheter replacement), (*P* < .05), PVI values were decreased in the same period but there were no statistical significance. The PI increases after peripheral plexus blockade and may be used as an indicator for successful block placement in awake patient. And also, it may be used as an indicator for catheter effectiveness after surgery. But PVI values cannot detect that kind of relation with nerve blockade.

## 1. Introduction

In the past years, many studies have been carried out with Massimo pulse CO-oximetry.^[1,2]^ Most of these studies include the plethysmographic variability index (PVI) and generally evaluate surgical blood loss and consider fluid therapy.^[3–5]^ Although the biggest advantage of Massimo pulse CO-oximeter seems to be noninvasively showing hemoglobin concentrations, it also shows the need for fluid dynamically with the help of a probe attached to the fingertip (Perfusion index, Plethysmographic variability index) among its advantages^[6]^ Perfusion index (PI), pulse It is calculated automatically with the oximetry sensor. PVI, on the other hand, occurs when vu varies with respiration, and blood flow is affected by heart rate and hemoglobin concentrations. In previous studies, PI was used in the evaluation of axillary nerve blocks in the upper extremity and sciatic nerve blocks in the lower extremities.^[7–10]^ Creating an appropriate and effective surgical block with traditional methods is usually evaluated with loss of pain or temperature sensation, and this requires patient cooperation. Many objective methods have been described to evaluate nerve blocks. In these interventions performed with local anesthetics, depending on the block of sympathetic nerve fibers; local vasodilation, increase in local blood flow and skin temperature occur.^[7,8]^ However, these clinical findings may not develop in the time required to evaluate adequate block formation and start the surgical procedure. PI calculated automatically by pulse oximetry; It is a parameter that indicates the circulatory state. It has been shown that the perfusion index is a useful method for evaluating the axillary nerve in the upper extremity and sciatic nerve block in the lower extremity in the area (finger) where the sensor is applied.^[7–9]^

In this study; we aimed to examine how sympathetic blockade (provided with axillary brachial block) will affect PI and PVI values and to show the possible correlation.

And also, PI and PVI values obtained from the measurement of peripheral perfusion with a finger sensor are a faster and more successful method compared to the traditional pinprick method in evaluating the success of block after axillary brachial plexus blockade.

## 2. Materials-methods

Our study was planned prospectively after the approval of Dokuz Eylul University clinical trials local ethics committee. One hundred ten patients over the age of 18 with American Society of Anesthesiologists I to II, who were planned to undergo upper extremity surgery with brachial plexus block and catheter placement, were included in the study. Patients who did not want to participate in the study, patients with known allergy to local anesthetics, pregnant patients, and patients with peripheral vascular disease were excluded from the study.

All patients included in the study were examined by an anesthesiologist before anesthesia. Following the appropriate fasting period, the patients were taken to the preparation room and routine monitoring (peripheral oxygen saturation, noninvasive arterial measurement of blood pressure, and electrocardiogram) were performed. Vascular access was established with a 20 gauge branule in the other arm, which did not undergo surgery. The patients were administered midazolam (0.05–0.1 mg/kg, Dormicum 5 mg/5 cc, Deva İlaç, Istanbul) for sedation before the procedure. Nasal oxygen was given at 2 to 4 L/minutes. In addition to routine monitoring, PI, PVI, Radical-7^TM^ finger pulse oximetry device (Massimo Corp, USA) in both fingers of 110 plastic surgery patients, who received an axillary brachial plexus catheter before surgery. After basal data recording, brachial plexus block and catheter placement procedure was started. The patient was in the supine position, the forearm was flexed and a 90-degree angle was given. The procedure site was sterilized, and the lineer ultrasound probe device was covered with a sterile sheath. Motor response of the terminal branches (median, ulnar, radial, and musculocutaneous) of the brachial plexus was evaluated by observing under ultrasound with 0.2 to 0.8 mA electrical stimulation, together with a 22 gauge 50 mm needle with a neurostimulator (StimuplexDig, B-Braun, Germany). For the block, it was prepared with 10 mL of 0.5% bupivacaine (Buvicaine 0.5%, 5 mg/mL Polifarma) and 5 mL of lidocaine (Jetmonal 2%, 20 mg/mL, Adeka, Turkey) and 5 mL of saline. A total of 20 ml of local anesthesia was administered to all patients. And the axillary catheter was placed in the radial nerve lodge and around the axillary artery.

The development of sensorineural block (inability to identify the cold application) in the motor block and related dermatomes was accepted as a successful block. Data recorded at baseline, during catheter replacement, after catheter replacement, and before surgery. All periods hemodynamic data, Visual Analog Scala (VAS), Ramsey Sedation Score and patient satisfaction score were collected.

## 3. Statistical analysis

In the statistical analysis of the collected data, continuous data will be expressed as median ± standard deviation and evaluated with the Mann–Whitney *U* test. Catagorized data will be expressed as a percentage value and evaluated with Chi-Square or Fisher exact test. The data will be analyzed with binary logistic regression analysis, axillary brachial block success as the dependent variable and all other independent variables. *P* < .05 will be considered statistically significant.

## 4. Results

Total 110 patients who developed successful block were included in the study. Two patients were excluded from the study because they were switched to general anesthesia. Patient characteristic data are presented in Table 1.

**Table 1:** Patient characteristics.

Characteristics	Values
Mean age (SD) in yr	38.28 (12.04)
Mean weight (SD) in kg	68.80 (7.34)
Mean height (SD) in cm	164.50 (9.41)
Sex, n	
M	57
F	53
Mean heart rate (SD) beats/min	64 (15.12)
Mean blood pressure (SD) in mm Hg	90.3 (8.72)

**Figure 1.**
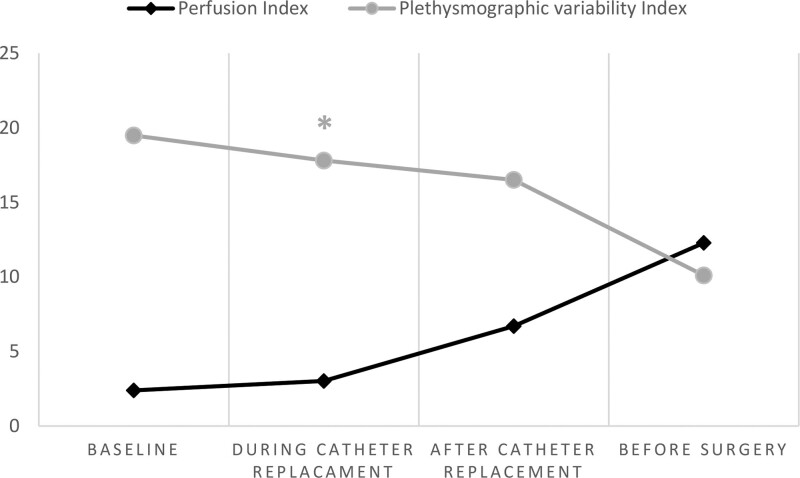
Perfusion index and Plethysmographic variability index values at different time intervals in patients. PI values in blocked arm increased after local analgesic application (during catheter replacement), (*P < .05), PVI values were decreased in the same period but there were no statistical significance. PVI = plethysmographic variability index.

In all 110 patients blocks were successfully applied, PI values in blocked arm increased after local analgesic application (during catheter replacement), (*P* < .05), PVI values were decreased in the same period but there were no statistical significance (Fig. 1).

**Figure 2.**
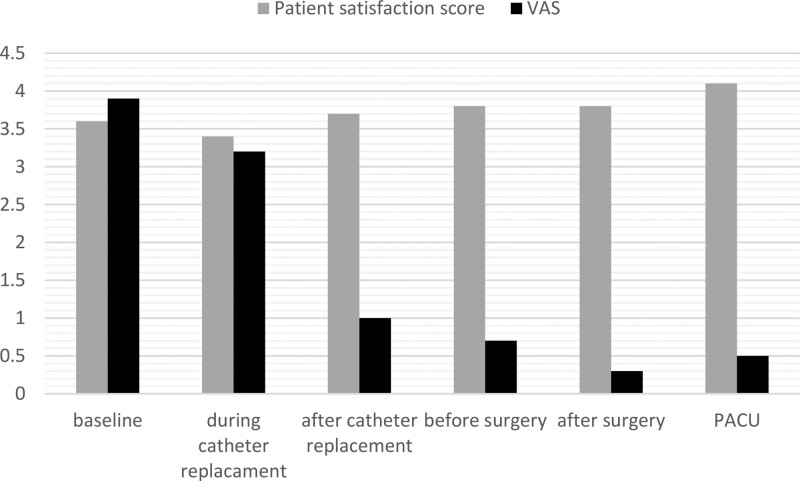
Patient satisfaction score and VAS values at different time intervals in patients. VAS = Visual Analog Scala.

When the patient satisfaction score and VAS, Ramsey Sedation Score values were evaluated at different time intervals; It was observed that patient satisfaction was quite high. Not surprisingly, VAS scores were found to decrease rapidly after catheterization and drug administration. In addition, the VAS scores of the patients remained at the lowest values after the end of the surgery and it was observed that the patient satisfaction was at the highest level (Fig. 2).

## 5. Discussion

In the surgical application with regional anesthesia, the degree of blockage should be known as motor and sensory. The degree of nerve blockade is the motor block provided with the sympathetic block. Since vasodilation caused by sympathetic blockade causes an increase in regional blood flow, an increase in the PI index is expected. Methods such as pinprick test, evaluation of temperature rise, evaluation of motor function or whether the patient has defined pain can be used to evaluate the success of the block. However, these methods can be subjective and patient-dependent variables. The formation of a successful block will prevent unnecessary drug administration to anesthesiologists, as well as increase the transition to general anesthesia and patient comfort.

In the literature, PI measurement was used to evaluate the successful application of peripheral plexus block. Abdelnasser et al^[11]^ in their study in which they evaluated the success of supraclavicular peripheral plexus block, they showed that PI measurement could be useful, similar to our results. PI was not only used to evaluate peripheral nerve blockade, but it was also shown that the change in PI was earlier than the change in skin temperature after epidural anesthesia.^[12]^ In another study conducted in patients who underwent epidural anesthesia, Galvin et al^[7]^ evaluated sympathectomy and showed that developing vasodilation increased the PI value. Klodell et al^[13]^ to evaluate successful sympathectomy in patients who underwent sympathectomy due to hyperhidrosis, they measured PI 2 ipsilateral upper extremities and showed that thoracic sympathectomy was effective. Sebastiani et al,^[14]^ who measured the change in PI in patients with interscalene nerve blockade, showed that the perfusion index is a sensitive parameter in demonstrating loss of vasomotor tone and successful sympathetic blockade after block. In the same study, they showed that administration of bolus iv fluid showed a greater change in PVI values in the non-blocked arm than in the blocked arm.

To confirm that vasomotor tone fluctuations induced by nociceptive stimuli alter PI, reduce the accuracy of PVI, and confirm the effects of surgical stimuli on PVI, Takeyama et al^[14]^ showed a significant increase in PVI before and after skin incision, and a negative correlation between PVI and changes in PI. showed a correlation. While PVI is routinely used for fluid response, it is important to pay attention to vasomotor fluctuations caused by nociceptive stimuli.

Bergek et al^[15]^ measured PVI, PI, and hemoglobin values in patients who underwent brachial plexus block, and showed that the PVI value decreased in the arm in which the block was applied. And it was shown that the PI value increased by 188% compared to the baseline value in the blocked arm. There was no significant change in PI value in the unblocked arm. It has been shown that this increase in PI value is due to vasodilation that develops after conduction block. They showed that the decrease in the PVI value also changed depending on the sympathetic blockade. We also used PI and PVI values in our study, and similarly, we found that there was a statistically significant increase in PI values due to sympathetic blockade, but it was not statistically significant, although there was a decrease in PVI values. In the literature, there are PI studies in the evaluation of peripheral block success. Interscalene block has been studied extensively in studies that are generally used to evaluate the success of upper extremity peripheral block.^[14,16]^ It has been shown that PI values are quite informative in the evaluation of pain and anesthesia.^[17]^ Similar to these studies, in our study with a larger number of patients, we showed that PI is an easily applicable and noninvasive technique to predict the success of block.

## 6. Conclusion

The PI increases after peripheral plexus blockade and may be used as an indicator for successful block placement in awake patient. And also, it may be used as an indicator for catheter effectiveness after surgery. But PVI values cannot detect that kind of relation with nerve blockade.

## Author contributions

**Conceptualization:** Hale Aksu.

**Data curation:** Hale Aksu, Dilek Omur.

**Formal analysis:** Elvan Ocmen.

**Funding acquisition:** Hale Aksu.

**Investigation:** Sezin Kizil.

**Methodology:** Hale Aksu, Dilek Omur, Sezin Kizil, Ayse Karci.

**Project administration:** Ayse Karci.

**Resources:** Sezin Kizil, Ayse Karci.

**Supervision:** Hale Aksu, Ayse Karci.

**Visualization:** Hale Aksu.

**Writing – original draft:** Hale Aksu, Ayse Karci.

**Writing – review & editing:** Ayse Karci.
